# The adenosine A2A receptor is associated with methamphetamine dependence/psychosis in the Japanese population

**DOI:** 10.1186/1744-9081-6-50

**Published:** 2010-08-30

**Authors:** Hideaki Kobayashi, Hiroshi Ujike, Nakao Iwata, Toshiya Inada, Mitsuhiko Yamada, Yoshimoto Sekine, Naohisa Uchimura, Masaomi Iyo, Norio Ozaki, Masanari Itokawa, Ichiro Sora

**Affiliations:** 1 Department of Biological Psychiatry, Tohoku University Graduate School of Medicine, Sendai 980-8574, Japan; 2 Department of Neuropsychiatry, Okayama University Graduate School of Medicine, Dentistry and Pharmaceutical Sciences, Okayama 700-8558, Japan; 3 Department of Psychiatry, Fujita Health University School of Medicine, Aichi 470-1192, Japan; 4 Department of Psychiatry, Seiwa Hospital, Institute of Neuropsychiatry, Tokyo 162-0851, Japan; 5 Department of Psychogeriatrics, National Institute of Mental Health, National Center of Neurology and Psychiatry, Tokyo 187-8553, Japan; 6 Division of Medical Treatment & Rehabilitation, Center for Forensic Mental Health, Chiba University, Chiba 260-8670, Japan; 7 Department of Neuropsychiatry, Kurume University School of Medicine, Kurume 830-0011, Japan; 8 Department of Psychiatry, Graduate School of Medicine, Chiba University, Chiba 260-8670, Japan; 9 Department of Psychiatry, Nagoya University Graduate School of Medicine, Nagoya 466-8550, Japan; 10 Schizophrenia Research Project, Tokyo Institute of Psychiatry, Tokyo 156-8585, Japan; 11 Japanese Genetics Initiative for Drug Abuse (JGIDA), Japan; 12 Current Address: Research Unit of Genome New Drugs, School of Pharmacy, Nihon University, Chiba 274-8555, Japan

## Abstract

**Background:**

Several lines of evidence suggest that the dopaminergic nervous system contributes to methamphetamine (METH) dependence, and there is increasing evidence of antagonistic interactions between dopamine and adenosine receptors. We therefore hypothesized that variations in the A2A adenosine receptor (*ADORA2A*) gene modify genetic susceptibility to METH dependence/psychosis.

**Methods:**

We first analyzed variations in the exons and exon-intron boundaries of the *ADORA2A *gene in METH dependent/psychotic patients. Then an association analysis between these single nucleotide polymorphisms and METH dependence/psychosis was performed using a total of 171 METH dependent/psychotic patients and 229 controls.

**Results:**

We found 6 variations, of which one single nucleotide polymorphism (SNP) was novel. Significant associations were observed between the allelic and genotypic frequencies of the Exon2+751 (rs5751876) SNP and METH dependence/psychosis. These associations were observed especially in females. In the clinical feature analyses, significant associations were observed between the SNP and the patient subgroup using METH alone (i.e., without concomitant use of other substances of abuse).

**Conclusions:**

These results suggest that the *ADORA2A *gene could be a vulnerability factor for METH dependence/psychosis, especially in females and/or in patients using only METH.

## Background

Methamphetamine (METH) is a psychomotor stimulant with high liability for abuse, and METH has become a very serious social problem not only in Japan [1] but also worldwide, including in the United States [2]. Use of METH induces a strong psychological dependence, and repeated usage frequently results in psychotic states, the symptoms of which are similar to those of paranoid-type schizophrenia [3,4]. Chronic METH abusers have been shown to have persistent dopaminergic deficits [5,6].

Amphetamines are thought to produce their stimulant effects mainly via the dopamine system [7,8], although other systems may also be involved. Dopamine D1 and D2 receptors exist as heterodimers with adenosine A1 and A2A receptors, respectively, which modulate their responsiveness [9,10], suggesting that responses to amphetamines may also be dependent on adenosinergic function.

Several lines of evidence suggest that adenosine A2A receptors (ADORA2A) play a role in inhibiting the effects of METH. ADORA2A antagonists have been shown to significantly increase the action of amphetamine-induced locomotor activity in mice [11] and to mimic the discriminative-stimulus effects of METH in rats [12]. ADORA2A agonists reduced amphetamine-induced locomotor activity in mice [11] and attenuated amphetamine-induced stereotypy in rats [13]. *ADORA2A *gene knockout mice showed attenuation in locomotor responses [14] and no development of locomotor sensitization to amphetamine [15]. These reports suggest the pharmacological potential of ADORA2A adenosinergic agents to modulate adaptive responses to METH exposure.

There have been no association analyses between *ADORA2A *gene polymorphisms and METH dependence/psychosis. In the present study, we analyzed all coding exons and exon-intron boundaries of the *ADORA2A *gene to reveal the variations in the Japanese population and examined the associations between novel and reported polymorphisms in the *ADORA2A *gene and METH dependent/psychotic patients in Japan.

## Methods

### Subjects

One-hundred seventy-one unrelated patients with METH dependence/psychosis (138 males and 33 females; mean age 37.5 ± 12.0 years) meeting ICD-10-DCR criteria (F15.2 and F15.5) were used as case subjects; they were outpatients or inpatients of psychiatric hospitals. The 229 control subjects (119 males and 110 females; mean age 41.2 ± 12.3 years) were mostly medical staff members who had neither personal nor familial history of drug dependence or psychotic disorders, as verified by a clinical interview. All subjects were Japanese, born and living in the northern Kyushu, Setouchi, Chukyo, Tokai, and Kanto regions. This study was approved by the ethical committees of each institute of the Japanese Genetics Initiative for Drug Abuse (JGIDA), and all subjects provided written informed consent for the use of their DNA samples for this research [3]. After informed consent was obtained, blood samples were drawn and genomic DNA was extracted by the phenol/chloroform method.

### Defining variants of the *ADORA2A *gene

Initially, DNA samples from 16 METH dependent/psychotic patients were used to identify nucleotide variants within the *ADORA2A *gene (NCBI accession numbers: AP000355 and NT_011520). Exons 1, 2 and exon-intron boundaries were amplified by polymerase chain reaction (PCR) using a thermal cycler (Astec, Fukuoka, Japan), and the products were sequenced in both directions using BigDye terminators (Applied Biosystems, Foster City, CA) by an ABI Genetic analyzer 3100 (Applied Biosystems). The primers used for amplifying exon 1 and its exon-intron boundaries were 1F and 4R, and those used for sequencing were 1F, 1R, 2F, 2R, 3F, 3R, 4F, and 4R (Table 1). The primers used for amplifying exon 2 and its exon-intron boundaries were 5F and 8R, and those used for sequencing were 5F, 5R, 6F, 6R, 7F, 7R, 8F, and 8R.

**Table 1:** Primers used in this study

Exon	Forward		Reverse	
Exon 1	1F:	GAGGTCCATTTGGATCCAGACCAT	1R:	TTCTCTCGCCAGGCCAACTTCTCA
	2F:	ACATCCTTCCACATCCGAGCTCCA	2R:	CAGGCCAGGTGTCAGCCTGAGGAT
	3F:	AGTCTCAGCGGGAATTCTATTGGA	3R:	GACGAAGCAGGCAATGAAGAGGCA
	4F:	TACATCACGGTGGAGCTGGCCATT	4R:	ACAGCTCCCTGCTAAGCCCAATGC
Exon 2	5F:	ACTACTCAATGACCATCTGGGCAT	5R:	TGATGGCCAGTGACTTGGCAGCAT
	6F:	TGCTGCTCATGCTGGGTGTCTATT	6R:	TCTTCTCCCAACGTCACTGGTCAA
	7F:	TTAGCCATGAGCTCAAGGGAGTGT	7R:	CTCTGGCACTGCTCTGTTACAACT
	8F:	TCACTCTCTGGCTGCTGGGTCTGC	8R:	GTCACAGTTCTGAGAAGGTAACAT

Genotyping of both Exon1+179 (rs13306114) and Exon1+219 was performed by PCR amplification using 3F and 4R primers followed by sequencing with 3F and 3R primers. Genotyping of IVS1+64 (rs13306116) was performed by PCR amplification using 4F and 4R primers followed by restriction enzyme *Bcn*I digestion (PCR-restriction fragment length polymorphism; PCR-RFLP). Genotyping of Exon2+751 (rs5751876) and IVS2+28 (rs34923252) was performed by PCR amplification using 5F and 8R primers followed by sequencing with 6F and 6R primers or 8F and 8R primers, respectively.

### Clinical category analysis

For the clinical category analysis, the patients were divided into two subgroups by four different clinical features. As some subjects had missing data for some clinical features, the sum of each subgroup was not total to 171.

(A) Latency of psychosis from first METH intake: less than 3 years (n = 64, average = 0.76y) or more than 3 years (n = 70, average = 9.4y). The course of METH psychosis varied among patients, with some patients showing psychosis sooner after the first METH intake, as previously reported [3,16]. Because the median latency was 3 years, this time point was used as the cutoff in defining the two groups. (B) Duration of psychosis after the last METH intake: transient (< 1 month) or prolonged (≥ 1 month). Some patients showed continuous psychotic symptoms even after METH discontinuation, as previously reported [3,16]. Psychotic symptoms disappeared in the patients with the transient-type psychosis within one month after the discontinuation of METH consumption and the beginning of treatment with neuroleptics. Patients with the prolonged-type psychosis had symptoms that continued for more than one month even after the discontinuation of METH consumption and the beginning of neuroleptic treatment. (C) Spontaneous relapse: present or not. It has been well documented that once METH psychosis has developed, patients in the remission phase are liable to spontaneous relapse without reconsumption [3,16]. (D) Multiple drug usage: mono-drug (use of METH without concomitant use of other drugs of abuse), or poly-drugs. Some patients consumed multiple drugs in addition to METH. They consumed at least either one of cocaine, marihuana, morphine, heroin, LSD, alcohol or paint thinner in addition to METH.

### Statistical analysis

The Hardy-Weinberg (HW) equilibrium of genotypic frequencies in each SNP was tested by the chi-square test. The allelic and genotypic frequencies of patients and control groups were compared using the chi-square test. The level of statistical significance was set at α = 0.05. The odds ratio (OR) and its 95% confidence interval (CI) were calculated as a measure of the association between Exon2+751 (rs5751876) alleles and METH dependence/psychosis. Haplotype frequencies were calculated by the Arlequin program available from http://anthropologie.unige.ch/arlequin[17]. A global test of differentiation among samples based on haplotype frequencies was performed using the Arlequin program.

## Results

### Analysis of *ADORA2A *gene variants

To identify polymorphisms in the *ADORA2A *gene, all coding exons and exon-intron boundaries were analyzed using genomic DNA from 16 Japanese METH dependent/psychotic subjects. Six single nucleotide polymorphisms (SNPs) were identified, of which one was novel (Exon1+219) (Table 2). Consistent with the previous study in Japanese samples [18], we could not find 405C/T, 432C/T, or 1018C/T polymorphisms. Two SNPs at Exon2+751 (rs5751876) and Exon2+1360 (rs35060421) were in linkage disequilibrium (LD) in the sense that the genotypic patterns of the 16 samples examined were the same. This LD has also been reported in a Caucasian population [19]. We chose Exon2+751 (rs5751876) to represent these SNPs. Exon1+179 (rs13306114), Exon1+219, IVS1+64 (rs13306116), Exon2+751 (rs5751876) and IVS2+28 (rs34923252) were chosen for further analyses.

**Table 2:** *ADORA2A *gene variants found in the Japanese population.

Location	Variants	rs#	Function	Reference
Exon1+179	C/T	rs13306114	untranslated	
Exon1+219	C/T		untranslated	
IVS1+64	G/A	rs13306116	intron	
Exon2+751	C/T	rs5751876	synonymous (Tyr- > Tyr)	1083C/T[30], 1976T/C[19]
Exon2+1360	T6/T7	rs35060421	untranslated	2592C/Tins[19]
IVS2+28	T/A	rs34923252	intron	

### Relationship between *ADORA2A *gene SNPs and METH dependence/psychosis

Association analyses between these SNPs in the *ADORA2A *gene and METH dependence/psychosis were performed using DNA samples from 171 METH dependent/psychotic subjects and 229 control subjects. The genotypic frequencies in these SNPs in controls were within the Hardy-Weinberg expectations. Significant differences were observed in both the genotypic (P = 0.018) and the allelic (P = 0.0057) frequencies of the Exon2+751 (rs5751876) SNP between METH dependent/psychotic patients and controls (Table 3). When Bonferroni correction was performed according to the number of SNPs examined, the corrected significance level was p < 0.01 (0.05/5 = 0.1). Significance was still observed in the allelic frequencies. Odds ratio of the allelic frequencies of the Exon2+751 (rs5751876) SNP between METH dependent/psychotic patients and controls was 1.50 (95% CI: 1.13 to 1.99).

**Table 3:** Genotype and allele frequencies of the *ADORA2A *gene SNPs in patients and controls.

SNP	Group	N	Genotype (%)			P	Allele (%)		P
Exon1+179 (rs13306114)			C/C	C/C	C/T		T/T	C	T
	Control	229	229 (100.0%)	0 (0.0%)	0 (0.0%)		458 (100.0%)	0 (0.0%)	
	METH	171	168 (98.2%)	3 (1.8%)	0 (0.0%)	0.132	339 (99.1%)	3 (0.9%)	0.154

Exon1+219			C/C	C/T	T/T		C	T	
	Control	229	228 (99.6%)	1 (0.4%)	0 (0.0%)		457 (99.8%)	1 (0.2%)	
	METH	171	169 (98.8%)	2 (1.2%)	0 (0.0%)	0.701	340 (99.4%)	2 (0.6%)	0.807

IVS1+64 (rs13306116)			G/G	G/A	A/A		G	A	
	Control	229	219 (95.6%)	10 (4.4%)	0 (0.0%)		448 (97.8%)	10 (2.2%)	
	METH*^1^	171	162 (94.7%)	8 (4.7%)	1 (0.6%)	0.504	332 (97.1%)	10 (2.9%)	0.663

Exon2+751 (rs5751876)			T/T	T/C	C/C		T	C	
	Control	229	70 (30.6%)	114 (49.8%)	45 (19.7%)		254 (55.5%)	204 (44.5%)	
	METH	171	35 (20.5%)	85 (49.7%)	51 (29.8%)	0.018	155 (45.3%)	187 (54.7%)	0.0057

IVS2+28 (rs34923252)			T/T	T/A	A/A		T	A	
	Control	229	181 (79.0%)	43 (18.8%)	5 (2.2%)		405 (88.4%)	53 (11.6%)	
	METH	171	144 (84.2%)	26 (15.2%)	1 (0.6%)	0.258	314 (91.8%)	28 (8.2%)	0.146

*1: METH sample: Hardy-Weinberg Equilibrium p = 0.0214

N: Number of samples

P: Significance values between METH samples and controls.

The SNPs having minor allele frequencies of over 5%, Exon2+751 (rs5751876) and IVS2+28 (rs34923252), were used for a global test of differentiation among samples based on haplotype frequencies using the Alrequin program. Significant association with METH dependence/psychosis was observed (P = 0.027).

Gender-dependent association analyses were performed. In female samples, the Exon2+751 (rs5751876) SNP showed significant associations with METH dependence/psychosis in genotypic (P = 0.0078) and allelic (P = 0.014) distributions (Table 4). When Bonferroni correction was performed by gender (i.e., using two categories: male or female), the corrected significance level was P < 0.025. Female samples still showed significance.

**Table 4:** Gender-dependent association analyses of the Exon2+751 (rs5751876) SNP in patients and controls.

Gender	Group	N	Genotype (%)			P	Allele (%)		P
Male									
			T/T	T/C	C/C		T	C	
	Control	119	31 (26.1%)	59 (49.6%)	29 (24.4%)		121 (50.8%)	117 (49.2%)	
	METH	138	27 (19.6%)	73 (52.9%)	38 (27.5%)	0.456	127 (46.0%)	149 (54.0%)	0.315

Female									
			T/T	T/C	C/C		T	C	
	Control	110	39 (35.5%)	55 (50.0%)	16 (14.5%)		133 (60.5%)	87 (39.5%)	
	METH	33	8 (24.2%)	12 (36.4%)	13 (39.4%)	0.0078	28 (42.4%)	38 (57.6%)	0.014

N: Number of samples

P: Significance values between METH samples and controls.

Subcategory analyses were performed on the clinical parameters (latency of psychosis, prognosis psychosis, spontaneous relapse, and multiple drug usage) on the Exon2+751 (rs5751876) SNP (Table 5). Nominal significance was observed in the transient course of prognosis of psychosis (P = 0.036), absence of spontaneous relapse (p = 0.046), and use of only METH (P = 0.0099), when they were compared to controls. When Bonferroni correction was performed according to the number of subcategories (2), the corrected significance level was p < 0.025. Significance was still observed in the subjects using only METH. But these differences were not observed between these patient subgroups.

**Table 5:** Association analyses between the Exon2+751 (rs5751876) SNP and clinically subcategorized METH subjects

Samples	Subgroups	N	Genotype (%)			P1	P2
			T	T/C	C		
Control		229	70 (31%)	114 (50%)	45 (20%)		
METH	Latency of Psychosis						
	< 3 years	64	15 (23%)	32 (50%)	17 (27%)	0.366	
	≥ 3 years	70	17 (24%)	30 (43%)	23 (33%)	0.068	0.663
	Prognosis of Psychosis						
	Transient (< 1 month)	91	21 (23%)	40 (44%)	30 (33%)	0.036	
	Prolonged (≥ 1 month)	56	11 (20%)	34 (61%)	11 (20%)	0.231	0.115
	Spontaneous Relapse						
	Not present	104	22 (21%)	50 (48%)	32 (31%)	0.046	
	Present	60	12 (20%)	31 (52%)	17 (28%)	0.167	0.905
	Multiple Drug Usage						
	None (METH use only)	52	6 (12%)	29 (56%)	17 (33%)	0.0099	
	Poly-drugs	112	26 (23%)	54 (48%)	32 (29%)	0.127	0.214

N: Number of samples

P1: Significance values between METH samples and controls.

P2: Significance values between the subgroups in each clinical category

## Discussion

We analyzed *ADORA2A *gene variations in a Japanese population and found six SNPs in exons and exon-intron boundaries. Significant associations were observed between the Exon2+751 (rs5751876) SNP and METH dependence/psychosis. The Exon2+751 (rs5751876) SNP showed significant associations with METH dependence/psychosis in female samples and with subjects using only METH.

This is the first report of an association analysis between *ADORA2A *gene polymorphisms and METH dependence/psychosis. The result that METH dependence/psychosis was significantly associated with *ADORA2A *gene polymorphism was in line with the findings in animal studies. In animals, the pharmacological effects of psychostimulants like cocaine and amphetamine were counteracted by ADORA2A agonists and potentiated by ADORA2A antagonists [20-23].

Exon2+751 (rs5751876) SNP showed a significant association with METH dependence/psychosis. As Exon2+751 (rs5751876) SNP is a synonymous variant, this SNP cannot include amino acid substitutions. However, we found one untranslated region SNP, Exon2+1360 (rs35060421, 2592C/Tins [19]), in LD with Exon2+751 (rs5751876). This LD was also reported in a Caucasian population [19]. While there is no evidence of functional alterations of these SNPs at this time point, one may hypothesize that untranslated region SNPs would change gene expression in a transcriptional and/or translational level, that lead to the pathophysiology of METH dependence/psychosis. Further analyses are necessary to study the functions of these SNPs or other SNPs in LD.

Significant difference was observed between the subgroup of patients using only METH and the controls in the variation of Exon2+751 (rs5751876) SNP, while no difference was observed between the subgroup of patients using poly-drugs and the controls nor within patient subgroups. The reason why the significant association was observed only in the patient subgroup using exclusively METH compared to controls is not known; however, further analyses in this patient subgroup could reveal the reasons for their predilection for METH alone. Actually, an association has been reported between the *ADORA2A *variants in the 1976C/T (Exon2+751) and 2592C/T(ins) (Exon2+1360) polymorphisms and the anxiogenic response to amphetamine in healthy subjects [24]. Hohoff and colleagues found that its effect on anxiety was stronger at the lower amphetamine dose and discussed that relatively small genetic effects are more relevant at lower doses of amphetamine and can be overcome by increasing the amphetamine dose [24]. Emotional alterations and/or the dose of METH use might be mediated by these SNPs and that will likely be one of the key to understanding METH dependence/psychosis.

We found a significant association between the female subjects with METH dependence/psychosis and Exon2+751 (rs5751876) SNP. These gender different associations between METH dependence/psychosis and SNPs have also been reported in several genes [25-28]. It has well been known and studied that male and female differ markedly with regard to their use of, and responses to, METH and related amphetamines [29]. Dluzen and colleagues summarized the data from published articles on gender differences in various parameters of METH use and responses and concluded that women seemed more dependent on and committed to METH but showed diminished (amphetamine-stimulated) dopamine responses and a decreased degree of toxicity [29]. Although larger replication studies need to be conducted on the association analysis between the SNP and METH dependence/psychosis in females, our results offer a potential means for understanding the differences between female and male in the progression to METH dependence/psychosis.

## Limitations

The limitation of our study is its moderate sample size, especially in gender-dependent association analyses and in clinical category analyses. Although the number of our study subjects is generally regarded as small for association studies, it is hard to obtain many patient samples due to the illegality of METH use in Japan. We therefore organized the Japanese Genetics Initiative for Drug Abuse (JGIDA) to collect samples. Based on these efforts, we could finally obtain this moderate number of patient samples. Nevertheless, our study needs to be reproduced in a larger sample size regarding the associations between these SNPs and METH dependence/psychosis.

## Conclusions

Our results suggest that the *ADORA2A *gene could be a vulnerability factor for METH dependence/psychosis, especially in females and/or in patients using only METH. Further investigations of the role of the *ADORA2A *gene in the development of METH dependence/psychosis are warranted.

## Competing interests

The authors declare that they have no competing interests.

## Authors' contributions

HK conceived of this study, genotyped samples, analyzed data and drafted the manuscript. HU organized the Japanese Genetics Initiative for Drug Abuse (JGIDA). HU, NI, TI, MY, YS, NU, MI, NO and MI collected genome samples and informed consents. IS supervised all managements, analysis and interpretation and revised the manuscripts to give final approval of the version to be published. All authors read and approved the final manuscript.
